# Hæmorrhage from Ulceration in the Throat, with Numerous Cases

**Published:** 1830-07-01

**Authors:** 


### XXXIX.

### HÆMORRHAGE IN CONSEQUENCE OF ULCERATION OR SLOUGHING OF THE THROAT.

Two of our contemporaries have published from time to time several cases of formidable, nay fatal, hæmorrhage, in consequence of ulceration of the throat. One rare case is of little importance, but when three or four of the same description are collated and compared, the subject assumes a commanding interest in the eyes of practical men. It has always been our object to act on this plan, and whenever it was proper or practicable we have endeavoured to bring into one consistent group such scattered specimens of unfrequent diseases, as would fail to strike the eye when single and apart, or if noticed would be glanced at as merely matters of curiosity. This is certainly a troublesome proceeding for the writer, but a very instructive method for the reader, and in our case we can affirm that the advantage of the one is never weighed in the scale with the convenience of the other. The first case to which we shall direct attention is one published by Dr. Watson, the able professor of clinical medicine in the London University.

### 1. FATAL HÆMORRHAGE FROM CYNANCHE TONSILLARIS.*

*Case.* Joseph Smith, æt. 26, a coachman, admitted into the Middlesex Hospital, Oct. 22, 1829, under the care of Dr. W.

"He complained of sore throat and inability to swallow. The external fauces were considerably swelled on both sides. He could open his mouth to a very small extent only; and he was unable to protrude his tongue, which was large and furred. It was, therefore, impossible to obtain a satisfactory view of the back part of the mouth; but the tonsils could be indistinctly seen very red and large, and on the left tonsil a white speck was visible. His breath was peculiarly offensive, and he complained of an unpleasant taste in his mouth. The pulse was about 90. The bowels were confined. He said that the soreness of throat had come on in the evening, five days before. He had been more than once wet through, in the preceding week; had felt unwell, and had some shivering, a day or two before the throat became affected.

Twelve leeches were immediately applied to the outside of the throat, beneath the angles of the jaws. Five grains of calomel were given in the evening, and the 'haustus sennæ compositus' of the hospital pharmacopœia the next morning.

---

* Med. Gaz. No. 57, Jan. 3, 1829.

On the 23d the outward swelling was not apparently diminished, but he said that he had experienced great and immediate relief from the leeches, and the bowels had been freely purged. He was directed to use a myrrh gargle, and to take ʒj. of the sulphate of magnesia in ℥iss. of the compound infusion of roses, every eight hours. From this time to the 5th of November there was but little variation in the daily reports, and not much change had occurred in the local symptoms. There was still much swelling of the external fauces on each side, and the same difficulty existed of obtaining a fair view of the tonsils. The patient was still unable to move his tongue, or to separate his jaws to the width of more than half an inch. During this period he had occasionally felt an obscure sensation of throbbing in the throat, and he sometimes complained of pain shooting from the throat into both ears. He was much distressed throughout by the accumulation of viscid and ropy mucus in the fauces, and was almost continually, whilst awake, trying to hawk up the mucus. The same offensive fœtor was present: this was in some degree corrected by a gargle containing the chloruret of lime, in the proportion of 15 grains to the pint of distilled water. The bowels continued freely open; the pulse was a little above 100, and of moderate strength and fulness; the skill cool. Leeches were repeatedly applied to the throat, always with relief to his uneasy sensations there, but never with any decided influence upon the external swelling, which remained hard and somewhat tender. A blister to the left side of the neck, and poultices, had also been applied, and the steam of warm water for some time diligently inhaled. No apparent benefit resulted from these measures. He obtained sleep and ease, after one or two very disturbed and restless nights, by the occasional exhibition, at bed-time, of half a grain of the acetate of morphia. He was able to swallow soft food."

On the 5th November, fluctuation being perceptible below the symphysis of the chin, a lancet puncture was made and much fetid pus discharged. On the next day he could open his mouth better, and no ulceration of the tonsils could be detected. On the 7th, at 9, a.m. florid blood to the amount of 12 or 14 ounces was discharged from the mouth, and coagulated into a clot intermixed with some portions of a curdy whitish substance. Infusion of roses with sulphuric acid directed against the back part of the mouth with a syringe arrested the bleeding, and obscure fluctuation being felt externally in the lower part of the throat, a puncture was made and some horribly fetid pus let out. At half-past 9, p.m. the hæmorrhage recurred, and a pint or more of florid blood issued in a small stream. The patient could open his mouth so imperfectly that the fauces could not be "mopped" by a sponge dipped in alum solution, and the latter was therefore syringed in, when the hæmorrhage ceased. At half-past 4, a.m. of the 10th, two quarts of blood lost, when the patient became faint. Dr. Southey and Mr. Mayo saw the patient when the bleeding ceased, and it was found impossible to ascertain precisely from what part it had proceeded. The opening externally made by the lancet, was surrounded by a gangrenous appearance for a quarter of an inch on every side. It was supposed that the lingual or some branch of it was opened, but on which side or where the consultants could not tell. It was determined therefore not to tie a carotid on a speculation, but to support the strength by bark and quinine. On the 9th the hæmorrhage did not return, but a quantity of dark coagulated blood was discharged from the external opening at the lower part of the throat, between which and the bleeding spot internally, it was obvious a communication must exist. The gangrenous appearance had not spread. On the 10th a small slough separated, and a puriform discharge had taken place from the external wound. On the 11th, he was reported as doing well; pulse 108; skin warm. From this time till the 21st he continued slowly to gain strength, and he was allowed a small quantity of mutton chop. Soon, however, after taking this, which excited coughing when swallowed, the hæmorrhage returned to a trifling degree, and at half-past 11, p. m. it recurred profusely, and did not cease till one or two pints of blood had been lost. At midnight Dr. Watson arrived and met Mr. Mayo in consultation. The patient was languid, chilly, weak, and pale, with a feeble pulse at 144;—a probe introduced into the wound passed towards the left side of the neck, on which the os hyoides could be felt quite denuded and rough. Mr. Joberns now joined the other gentlemen, and it was determined to tie the left carotid artery. In moving the patient for this purpose the hæmorrhage recurred afresh, and although Mr. Mayo was able, by introducing his finger into the wound, to compress the artery between his finger and thumb, the patient sank, became convulsed, and presently expired. The mode of death was by asphyxia and not by syncope.

"This conclusion, which the symptoms attending the act of dissolution had not left doubtful, was confirmed upon the examination of the body on the 23d, thirty-seven hours after death. The carotid artery had been previously injected, and its branches were filled with wax: these branches were severally traced. It was found that an abscess had existed, which opened internally behind and below the left tonsil, and nearly opposite to the epiglottis; through this outlet the bleeding into the mouth had taken place; the wall of the abscess where it opened internally was very thin. This abscess ran along the left side of the larynx, leaving the os hyoides rough and bare, and terminated externally by the opening already described, and which was made by the lancet. The facial and lingual branches of the carotid arose (as they are known frequently to do) by a common trunk. The lingual branch was traced to the situation of the abscess, where it terminated by an open mouth, about which the matter of the injection was found extravasated; on tracing the same artery beyond the abscess, it was seen to be quite empty. There could be no doubt that this was the artery from which the hæmorrhage had proceeded, and that it had been divided by ulcerations at the place where the abscess was situated. On slitting up the trachea, bronchi, and their branches, they were found to contain coagulated blood; there was a firm clot in the larynx; and another, composed partly of tenacious mucus, in which the coagulum of blood was mixed and entangled, remained at the summit of the fauces. The lungs were large and distended, and blood could be traced through many of the bronchial ramifications into the very air vesicles; this was more the case in some parts of the lungs than in others. A section of those parts where it was most evident presented just such appearances, but smaller and more numerous, as are seen in what Laennec has called 'pulmonary apoplexy.' On the external surface of the lungs, as is frequent in that disease, some well defined spots, of an uniformly dark colour, were visible; upon cutting into these they were found to have been produced by a sanguineous engorgement of the extreme vesicular branches of the air tubes, the boundary depending apparently on the extent of the lobules in the several cases. In some parts of the lungs this appearance did not exist—in those parts where it occurred, it was partial; blood had been forced to the extremities of some air tubes, and not of others. The blood in the blood-vessels was fluid, and escaped before the condition of the cavities of the heart, in regard to their contents, was ascertained. No vestige of ulceration could be perceived on either of the tonsils."

We have one remark to make on this interesting and really melancholy case, and that is an expression of something like surprise, at no medicine of astringent or styptic properties having been administered internally. Dr. Watson is well aware of the powers of the acetate of lead in checking hæmatemesis and hæmoptysis, and that too when the hæmorrhage is of a formidable character. Yet the acetate would appear to have been withheld altogether, nay it is not even mentioned, in the present instance. There is another remedy of a different character, which is undoubtedly possessed of considerable powers as an internal astringent, we mean the quack medicine which bears the name of the styptic of the Chevalier Ruspini. What it is we do not know, and as practical men we do not care, but we are certain that it is a valuable agent in arresting hæmorrhage. We have witnessed its powers on several occasions, and we have heard that in a severe case of hæmoptysis which lately occurred at St. George's Hospital, it proved effectual in stopping the discharge, after alum, acids, and the acetate of lead had failed. We are rather surprised that no medicine whatever of this class was exhibited by Dr. Watson in the present case, but we dare say he could offer a satisfactory explanation of the omission.

II. SLOUGHING OF THE THROAT—HÆMORRHAGE—SUCCESSFUL LIGATURE OF THE CAROTID.*

*Case.* This is detailed by Mr. Luke, of Broad Street Buildings, Surgeon to the London Hospital. The result was more fortunate than in the preceding case.

T. B. ætatis 45, a tall, rather muscular man, of sanguineous temperament, captain of a coasting vessel, trading between Cornwall and London, while in the former place, was stung by a wasp on the wrist, which became much inflamed, attended by a pustular eruption around the part. Livid red blotches, about three days after the sting, appeared on the trunk and extremities, with fever, neither of which created any alarm. With these upon him he went on board his vessel, bound to London. On his passage he had the misfortune to take cold, and was affected with sore throat, requiring confinement to his cabin. In a week he arrived in London, much worse, at which time he was visited by Mr. Gayton. The soreness, however increased, and the difficulty of swallowing was very considerable. He experienced much pain, particularly in the left side, where he was convinced a 'gathering had formed.' His opinion was confirmed on Sunday, Sept. 27th, by the bursting of an abscess, with partial relief. Together with the matter he passed about six ounces of blood by the mouth. He was still sensible of another gathering lower in the throat than the first on the same side; and exhausted as he was by disease, he began to entertain apprehensions for his safety. On Sept 29th he was brought on shore, and took up his residence with a friend in the neighbourhood of Mr. Luke's house. On the eve of this day, the second abscess burst, and shreds of slough came away with the matter: by this he was much relieved, and slept the greater part of the night.

"Sept. 30th, three weeks from the commencement of his illness, I was called to him about four o'clock in the morning, in consequence of his having lost a large quantity of blood. He had been awoke by something flowing from his throat, which proved to be blood. When I arrived he had already lost about half a wash-hand basin full of blood; and shortly after he vomited more than two pints of coagula, making altogether between four and five pints lost in about half an hour. I found him in the greatest state of exhaustion; his pulse was scarcely perceptible and very rapid. There was extreme paleness of the lips; the eye sunk in the orbit; a clammy sweat upon the skin; inclination to vomit; and he could not speak louder than in whispers. On attempting to examine the throat, I could see nothing behind the soft palate, to which were adhering shreds of coagula. In a short time he began to revive, and his pulse became more perceptible. Ordered one grain of the acetate of lead, with a quarter of a grain of opium, every two hours; goulard lotion, with equal proportion of spirit, to be applied to the throat; the head to be elevated on a pillow, to be kept perfectly quiet, and abstain from swallowing as much as possible."

The faintness passed away, and at 12 o'clock he was much revived. He had an anodyne at night, and, next day, was much improved in appearance; the blotches were clearly those of the purpura hæmorrhagica. Ordered to con-

* Med. Gaz. No. 106. Dec. 12, 1829.

tinue as before, with the addition of beef tea. On the third, at 4, p.m. Mr. Luke was again summoned on account of a return of the bleeding, which, however, had ceased before his arrival, the patient having lost, in a quarter of an hour, between three and four pints of blood. He did not appear much exhausted, but complained in a whisper of the left side of the os hyoides, as the seat of pain, and the spot from which he had felt the jet issue. To continue the lead with the addition of half a grain of powdered digitalis, every two hours.

"Oct. 4th.—At 4 a.m. bleeding again returned. From the account I received I expected to find my patient dying or dead. I found him in the greatest possible state of exhaustion; faint to nausea; the pulse with difficulty to be felt, and very rapid; the breathing laborious, and extreme paleness of the countenance. He was sensible, but apparently indifferent to surrounding objects. He had lost at this bleeding more than three pints of blood. It seemed almost certain that he must die. After a short time, however, he began to revive; the pulse became more distinct, and breathing more free, but the powers of life were so far reduced that another bleeding would inevitably prove fatal. I therefore determined to tie the carotid artery on the left side, that being the trunk which the circumstances of the case indicated to be the source of the bleeding vessel. To obtain the advantage of day-light, the captain was seated on a chair near a window. Before, however, he was arranged for the operation, his face became convulsed, the pupils of his eyes dilated; his head fell upon his shoulders; and his pulse and respiration ceased. In this state he was hurried back to bed, with the impression upon my mind that he was past hope. A few minutes shewed that he had only fainted, and he soon revived. His head was then laid over a pillow at the foot of the bed, and as the room was dark, I was obliged to proceed by candle-light. An incision of about three inches through the skin, exposed the platysma hyoides, which was divided along the inner border of the sterno-mastoideus muscle. This being drawn to one side exposed the omo-hyoideus crossing the sheath of the vessels, which was then cut through. The carotid could be very indistinctly felt pulsating in its sheath, into which last I made a small opening; a director was then introduced to detach the artery from the accompanying nerve and vein. This being done, a needle, armed with a double ligature, was carried around it from the outside, without bringing into view either the nerve or the vein. The ligatures were separated, and tied about half an inch apart, and the wound closed with plaister. On being questioned, he said he did not experience any unusual sensation when the ligature was drawn tight. His pulse was very weak, and beating 120 in a minute. The pupil of the left eye more dilated than the right. He was kept in the same position as during the operation, except that his head was not so much extended. Ordered beef-tea and light drinks."

No hæmorrhage took place till the 7th, when the wound was dressed, and found to adhere for half its extent. He had been feverish during the preceding night, and in the evening he spat up about an ounce and a half of saliva, tinged with blood. Salts and senna immediately—infusion of roses, with gtt. xv. of Tr. Digital. every six hours —syrup of poppies for the uneasiness of the throat. He passed a restless night, but was better next day; on examination, a slight fulness only could be seen on the left side; he swallowed with ease. Oct. 11th. Again fever and irritation of the throat; has spat up 2 ounces of blood; bowels confined. To repeat opening draught as occasion requires. He left off the digitalis on the 13th, in consequence of head-ache; the pupil of the left eye dilated, but vision as good as with the right. The bowels required to be kept regular, as fever otherwise ensued and a gum-boil, filled with congealed blood, formed over the incisor teeth. The ligatures were twisted, to expedite their separation. On the 26th October, the twenty-second day from their application, the ligatures were removed. After this day he went down stairs daily, and is now enabled to call on his friends and transact business. He has no unusual sensation, but is weak and soon fatigued. Pulsation has returned in the arteries above the os hyoides, but not in the trunk between this bone and the site of the ligatures. The wound was not quite healed when he left town for the country.

### III. HÆMORRHAGE FROM AN ULCER IN THE FAUCES—SUCCESSFUL LIGATURE OF THE COMMON CAROTID ARTERY.*

*Case.* J. Webb, æt. 23, was admitted into the Middlesex Hospital on the evening of the 18th of October. Those who brought him stated, that he had suddenly lost a considerable quantity of blood from an ulcer in the fauces; but the hæmorrhage was now stopped, and no apprehension was entertained of its immediate return. At 9 a. m. next morning, the bleeding broke out afresh, but was stopped by the house-surgeon's making pressure on the carotid artery of the affected side. On Mr. Mayo's arrival at the Hospital, he found the patient pale, bloodless, and faint to the last degree.

"On examining the fauces, I saw a ragged clot of blood adhering to the right side of the pharynx, while the left tonsil and adjacent surface appeared clear and healthy. I proceeded, therefore, without loss of time, to tie the right common carotid in the middle of the neck. Scarcely a drop of blood flowed from the incision made for this purpose; the pulse in the artery, when it was exposed, was exceedingly feeble; the internal jugular vein lay shrunk and collapsed.

After the operation the patient several times fell into an alarming state of faintness; but having taken some brandy, some Spiritus Ammoniæ Aromaticus in water, and some strong and spiced broth, he gradually rallied.

A few minutes after the artery was tied, I inquired of this patient whether he saw equally well with both eyes. He closed his eyes alternately, to ascertain the fact; and remarked, that his vision with the right eye was dim and obscure, while he saw distinctly with the left. At this time I could perceive no pulse in the right temporal artery. During the afternoon, distinctness of vision with the right eye returned; at the same time the pulsation of the right facial artery could be felt, though it was much less forcible than that of the left. The patient stated that he felt considerable throbbing in the left side of the head. He dozed much during the day, and slept well the following night.

The next morning an attempt was made to learn the history of his indisposition. The account which he then gave, however, and has since repeated, is very imperfect. He states that, for the last four months, he has had a sore throat; that, three months ago, spots broke out upon his chest and legs; that he took pills for these complaints during the space of three weeks, and that his mouth was affected about a fortnight; that, under this treatment, the spots upon his chest went away; that, for the last six weeks, he has taken no medicine, but has used a gargle; and that his throat he has latterly conceived to be getting better. The spots on the legs and thighs have left superficial ulcers, (which, when they were shown for the first time some days after his admission, were healing.) He states that he had a gonorrhœa a year ago; sores on the private parts never."

On the 20th, the following was the appearance of the throat:—the right margin of the uvula and edge of the right side of the soft palate were in a state of ulceration; the right tonsil was entirely destroyed with the posterior arch of the palate; the right side and posterior surface of the pharynx were ulcerated and covered with viscid puriform secretion; and at one part a portion of ashy slough adhered to the surface. Bark and acid with a chloride of lime gargle were prescribed, but no material change taking place, the cinnabar fumigation was directed on the 31st. On the following day the whole of the ulcerated surface was covered with florid granulations. On the 3d Nov. the 15th day after the operation,

* Med. and Phys. Journal, Dec. 1829.

the ligature came away from the artery, and the ulcer of the pharynx had begun to cicatrize. On the 16th the right side of the pharynx and palate had cicatrized. The ulcer which remained at the back of the pharynx was an inch in length, half an inch in breadth, yellow, and at one part deeply excavated. Blue pill internally, and a mercurial ointment to the ulcer were employed, healing went on rapidly, and on the 22d Nov., the last date of report, there was every reason to believe that a few days more would complete the cicatrization.

"Till the ulcer in the throat assumed a healthy appearance, I felt considerable anxiety lest the hemorrhage should return; and I regretted that, instead of tying the common carotid, I had not tied the internal and external carotids separately at their origin. This operation (which, if I were again called to a similar case, I should probably adopt,) is calculated to give the patient an additional security against a recurrence of hemorrhage; inasmuch as it would cut off, not merely the *direct flow* of blood upon the ulcerated artery, but also the *principal anastomotic supply*.

In these remarks, I take it for granted that the hemorrhage in the case described proceeded from a branch of the *external* carotid. They would not, it is evident, apply if the ulcerated vessel were the *internal* carotid: in that case, it is to be feared that nothing would save the patient.

In the present instance, I am inclined to suppose that the ulcerated artery was the *lingual*, for the following reasons: 1. The patient tells me that the soreness in his throat, which he latterly experienced, seemed to him deeply seated within the angle of the jaw; and thus referred to the exact situation of the lingual artery, which as regards the cavity of the fauces, is singularly exposed and superficial at this part. 2. In a case attended by Dr. Watson, in some respects very parallel to Webb's where the patient died through hemorrhage into the fauces from ulceration, the artery which bled was proved, by dissection, to be the lingual artery."

Cases similar to the preceding are of rare occurrence. In some the first hemorrhage is fatal; in others, the ulcerated artery, having bled for a time, spontaneously closes, and the bleeding does not recur: in others, the patient is carried off by a return of hemorrhage. In the case of Webb, there can be little doubt that the latter result would have ensued if the artery had not been tied. I believe the case to be the first of the kind in which this operation has been performed: it is extremely gratifying to me to have to state that the practice has proved successful."

The successful issue of the case is a flattering panegyric on the bold and decisive operation performed by Mr. Mayo. But we have the same remark to make on the treatment which we made on that adopted in the case of Dr. Watson, and we cannot but regret that none of the usual medicinal means employed for checking hæmorrhage, were resorted to previous to the use of the knife. The patient was in the house for nearly, if not quite, twelve hours before a recurrence of the bleeding took place, and during that time the employment of the acetate of lead, &c. would have done no harm, but possibly might have been productive of good. These observations can hardly apply to Mr. Mayo, for the patient was admitted at a late hour in the evening, and probably was not seen by Mr. M. till the next morning's catastrophe, which required more prompt and energetic measures.

### IV. Hæmorrhage from Sloughing Ulcers in the Throat successfully treated without an Operation.*

*Case.* Wm. Stennet was admitted into Lazarus's ward, Bartholomew's Hospital, Oct. 9th, 1829, in a very debilitated state, with a large sloughing ulcer occupying the whole of the back of the fauces, and extending to the edges of the soft palate and uvula. He

* London Med. Gaz. No. 113, Jan. 30th. 1830.

stated that at the latter end of April he was affected with an ulcer on the inner membrane of the prepuce, near its junction with the corona glandis. The sore was not excavated, but notwithstanding was very red and hard at its base. He took some mercury, and the sore skinned over without his mouth being affected. About the end of July a bubo appeared in each groin, which suppurated and burst spontaneously. On the 27th of Sept. his throat became sore, and gradually got worse until he was admitted into the hospital. At this time there was an open sinus in the groin; the cicatrix of the original sore was hardened; and, in addition to his sore throat, there were several dark-colored tubercular eruptions on his forehead. He was in so weak a state that mercury was not at first resorted to. He was ordered a very strong preparation of the red Jamaica sarsaparilla three times a day, and the throat was painted over with the linimentum æruginis. He was also directed frequently to wash the throat by throwing a stream of water from an elastic gum-bottle upon the ulcer, while he held his mouth open over a basin—a simple plan of cleansing a throat, which has been found far more efficacious than gargling.

On the 21st, as he did not appear to gain any ground, and the sloughs were deeper and very extensive, and his stomach rejected the sarsaparilla, he was ordered Quinæ Sulph. gr. ij. ter die ex infus. rosæ. Vini. Rubr. Oss. quotidie, and a strong solution of Nitrate of Silver was applied to the throat.

23d.—His general health was improved, but the sloughing still extended. He was ordered to fumigate with cinnabar night and morning. The second application produced such violent bronchial irritation that it was necessary to bleed him, and to desist from the fumigation.

By the 27th he had recovered from the bronchial affection, and his throat was much cleaner. The pure nitrate of silver was applied over the surface; milk and arrow-root diet, and sarsaparilla, were again resorted to, and he was removed into a *clean* ward. His general health improved, his throat began to granulate, and he was apparently going on well until the 25th of Nov. when the remaining portion of the uvula sloughed away, and the whole of the fauces again assumed a very threatening aspect. As the local application of the mercury had before benefitted it, the lotio flava was directed to be applied to the throat; and he was ordered to take Hydr. Oxymur. gr. $\frac{1}{8}$ ter die.

"On the 4th of Dec. as the throat was not improved, he was again ordered to employ the fumigation, with greater precaution than on the former occasion. During the night he felt a peculiar sensation in his throat, requiring him frequently to swallow. At 4, a.m. he vomited up nearly three pints of blood, and became alarmingly faint. The house-surgeon, Mr. Chapman, was sent for, who ordered him Plumbi. Acet. gr. j. Opii. gr. ss. 4tis horis, and directed him to take every thing quite cold. The bleeding did not recur before I visited him at half-past 12. He was then in a most alarming state; his pulse so feeble that it could hardly be distinguished; and his whole body bathed in a cold clammy sweat. It was quite obvious that a recurrence of bleeding must prove speedily fatal. I had just heard of Mr. Mayo's successful operation, and should have been disposed to give the patient the chance of success from the same means, but it was quite impossible to determine from which side the bleeding took place, so very extensive was the sloughing in every direction. Under these circumstances he was directed to take Alum. gr. x. ex Inf. Rosæ. ℥iss. c. acid. Sulph. dilut. ♏x. et Træ. Opii. ♏v. 4tis horis. He was kept in a state of the greatest quietude; fed entirely on iced fruits and milk; and most narrowly watched. Without detaining your readers with too minute a detail of the case, suffice it to say, no return of bleeding took place. In a week he was much recovered in his strength, though very feeble. As the throat was still in a very bad state, and the sores on his head were spreading, the nurse was desired to rub Ung. Hyd. fort. ʒj. night and morning, into the axilla. The mercury speedily began to have a most beneficial effect; the sores gradually improved, and are now nearly healed; his strength and general health have also improved in proportion. He has since left off the mercury, and has resumed the sarsaparilla."

Mr. Earle, under whose care the preceding case turned out so happily has added some observations that are worthy of attention. They certainly conspire with the issue of this case and of another to which Mr. Earle alludes, to support the suggestions we have offered.

"I have stated that I should have been induced to have tied the trunk of the lingual, or the external and internal carotids, in this case, if it could have been clearly ascertained from which side the bleeding took place. I need hardly add, that if such an operation had been performed, and the patient had recovered, it is probable that the recovery would have been attributed to the employment of the ligature. It is on this account that I think it due to the profession to publish the case; at the same time I wish it to be distinctly understood that I do not pretend to offer an opinion respecting Mr. Mayo's or Mr. Luke's cases. I am desirous of taking this opportunity of concurring in opinion with Mr. Mayo in the propriety of tying the external and internal carotids separately in all such cases as may require the ligature of these vessels; but I should prefer tying the trunk of the lingual where such an operation could be effected. In Mr. Luke's case it is obvious that the circulation continued through the bleeding vessel, as several slight returns of arterial hæmorrhage took place. It is probable that in this case, if the force of the heart and arteries had been greater, the operation would have failed, from the collateral circulation.

A case in every respect similar to Stennett occurred in Sewell's ward in the autumn of 1828. In this case a young, very delicate female, had repeatedly extensive hæmorrhage from foul ulcers occupying the whole fauces. The bleeding was successfully arrested by the same means as were employed in Stennett's case—namely, large doses of Sulph. Aluminis in infus. Rosæ, and feeding the patient on iced milk and fruits."

By the way, neither Mr. Earle nor Mr. Luke have alluded to another very powerful measure in arresting hæmorrhage, we allude to venesection. If a patient were swooning or sinking from violent bleeding, no one would of course be so mad as to open a vein in his arm, but generally the fatal loss of blood is preceded by several slighter ones, and that is the time for moderate bleeding from the arm, aided by acids or astringents, cool air, cool drinks, position, local pressure, and the other items of the anti-hæmorrhagic regimen. If these fail, the surgeon has the cases of Mr. Mayo and Mr. Luke, as precedents to guide him. The subject is very interesting, and the facts we have now brought together are calculated to be instructive.

